# Identification of Site in the *UTY* Gene as Safe Harbor Locus on the Y Chromosome of Pig

**DOI:** 10.3390/genes15081005

**Published:** 2024-08-01

**Authors:** Xiaomei Chen, Guang Yang, Pengyun Ji, Guoshi Liu, Lu Zhang

**Affiliations:** 1 State Key Laboratory of Farm Animal Biotech Breeding, Frontiers Science Center for Molecular Design Breeding, College of Animal Science and Technology, China Agricultural University, Beijing 100193, China; 13413663307@163.com (X.C.); yangguangimu@163.com (G.Y.); jipengyun@cau.edu.cn (P.J.); gshliu@cau.edu.cn (G.L.); 2 College of Animal Science and Technology, Sanya Institute of China Agricultural University, Sanya 572025, China

**Keywords:** pig, *UTY*, genomic safe harbor, CRISPR/Cas9, homology-mediated end joining

## Abstract

Genomic Safe Harbors (GSH) are loci used for the insertion of exogenous genetic elements, enabling exogenous gene expressing predictably without alterations of the host genome. These sites are becoming increasingly important as the gene editing technologies advance rapidly. Currently, only a few GSHs have been identified in the pig genome. In this study, a novel strategy was demonstrated for the efficient insertion of exogenous genetic material into the third exon of the *UTY* gene on the Y chromosome using CRISPR/Cas9-mediated homology arm-mediated end joining. The safety of the locus was verified according to the proper expression of the inserted *EGFP* gene without altering the expression of *UTY*. This approach enables the integration and expression of the exogenous gene at this locus, indicating that the *UTY* locus serves as a genomic safe harbor site for gene editing in the pig genome. Located on the Y chromosome, this site can be utilized for sex-biased pig breeding and developing biomedical models.

## 1. Introduction

With the rapid advancement of gene editing technologies, many gene-modified pigs have been successfully generated and applied in medical research or livestock breeding. Recently, genetically modified pigs have served as kidney, heart or other organ donors for human xenotransplantation and have been tested in several preclinical research studies [1,2]. Meanwhile, pigs with a resistance to PRRS (Porcine Reproductive and Respiratory Syndrome), CSFV (Classical swine fever virus) and TGEV (transmissible gastroenteritis virus) were produced by varied genetic modification strategies [3,4,5]. The latest advancements, that the gene-edited ‘GalSafe’ pigs are allowed to entry into the market in the United States [6], make it possible to utilize genetic modifying methods in the wild range of the livestock industry.

Gene-editing nucleases, such a Zinc Finger Nucleases (ZFNs) [7,8], Transcription Activator-Like Effector Nucleases (TALENs) [9,10] and the clustered regularly interspaced short palindromic repeats (CRISPR)/CRISPR-associated protein 9 (Cas9) system [11,12], facilitate the insertion and integration of exogenous genetic elements into the genome by inducing locus-specific DNA Double-Strand Breaks (DSBs). In the past, transgenic vector construction primarily relied on homologous recombination (HR), which is only active during the late S and G2 phases of the cell cycle, resulting in quite a low efficiency in animal embryos and in vivo tissues [11]. Non-Homologous End Joining (NHEJ) is another method to repair the DSBs, but it can induce random integration of the exogenous DNA fragments and various mutations [13]. These make it challenging to generate targeted proteins from the fusion genes [14]. In recent years, Microhomology-Mediated End Joining (MMEJ), which is active during the early S and M phases, has emerged. MMEJ can precisely mediate the integration of exogenous genetic marital into the targeted chromosome, but it exhibits a lower knock-in efficiency in cells [15,16]. Meanwhile, Homology-Mediated End Joining (HMEJ) has been described a novel method for transgenic vector construction. HMEJ combines HR and a new way to repair DSBs involving homology-mediated end joining for targeted integration, resulting in high integration efficiency in both embryos and non-dividing cells [16].

Genomic safe harbors (GSHs) are loci in the genome that allow exogenous genetic elements to integrate and ensure their expression as predicted without causing alterations of the host genome [17,18]. Currently, only three loci, *pRosa26* [19], *pH11* [20] and *pifs501* [21], have been identified as safe harbor loci in the pig genome for the insertion of exogenous genes and they have been successfully used in the production of transgenic pigs [19,21,22]. Meanwhile, the sites in the *pGAPDH* [23] and *pACTB* [24] genes have also been considered as potential GSHs in the pig genome, but these loci have not yet been utilized for the transgenic pig production. Furthermore, all these GSHs are all located on autosomes. Identifying additional GSHs is crucial due to the scarcity of GSH loci for the knock-in of exogenous genes in transgenic pig production. There is an urgent need to identify a gene sanctuary locus located on the sex chromosome for conducting research on pig sex differentiation and gender selection.

The production of domestic animals with a desired gender is essential for the livestock industry, and gene editing has become a powerful method to accelerate genetic selection [25]. The ubiquitously transcribed tetratricopeptide repeat gene Y-linked (*UTY*) was identified as a single-copy gene located on the Y chromosome of animals, encoding a histone demethylase [26]. Recently, Cas9-EGFP was integrated into the mouse *UTY* locus by HR to produce the XY^Cas9^ mouse model [25]. Subsequently, a population of mice with 100% monosexuality was obtained by crossing with the *H11^TOP^*^1^ mouse strain [25]. Therefore, the sites in the *UTY* gene in pigs’ Y chromosome may serve as safe harbor loci for gender-biased genetic modification.

This study aimed to identify the safe harbor sites on the sex chromosomes for conducting sex-specific gene editing in pigs. The experiments were performed to verify whether the loci of *UTY* on the porcine Y chromosome can serve as a safe harbor loci for exogenous gene integration for gender-biased genetic modification. By constructing px459 targeting vectors, the sgRNA with a high targeting efficiency was identified, and then, the *EGFP* fragment was integrated into the pig genome using CRISPR/Cas9-mediated HMEJ. The current findings provide a strategy for integrating exogenous genes into the porcine genome and confirm that the loci of *UTY* can serve as safe harbor loci to produce gender-biased genetically modified pigs.

## 2. Materials and Methods

### 2.1. Plasmid Construction

The *UTY*-specific single guide RNA (sgRNA) was designed using the online website https://cctop.cos.uni-heidelberg.de/, accessed on 6 September 2023, where the oligonucleotides encoding the sgRNA are annealed and assembled into the linearized px459-SpCas9-NG vector (Addgene Plasmid #171370, HedgehogBio, Shanghai, China). The oligonucleotides encoding the sgRNA were denatured using a PCR machine with the following program: 37 °C for 5 min, followed by 95 °C for 30 min and, finally, incubated at room temperature (25 °C) for 1 h. Subsequently, the annealed oligonucleotides were ligated to the BbsI-digested px459 vector (HedgehogBio, Shanghai, China). The ligation mixture was then transformed into Escherichia coli Top10 competent cells (Sangon Biotech, Shanghai, China). The sgRNA oligonucleotide sequences are listed in Supplementary Table S1. Then, the annealed oligonucleotides are ligated to the BbsI-digested px459 vector, followed by transformation of the ligation mixture into Escherichia coli Top10 competent cells (Sangon Biotech, China). The sgRNA oligonucleotide sequences are listed in Supplementary Table S1. PUC19 was used as the backbone to construct the donor vector pUC19-F2A-EGFP (Figure S1). The homologous arms, *EGFP* sequence, and F2A sequence are connected together using standard overlap PCR, and then inserted into the pUC19 vector obtained from Addgene (Addgene Plasmid #50005, HedgehogBio, Shanghai, China). The homologous arms for end joining in the pUC19 locus extended up to 800 bp on both the left and right sides.

### 2.2. Cell Culture and Transfection

The swine testis (ST) cells were optimal for examinations, as the *UTY* gene is located on the Y chromosome. Pig ST cells (Wuhan Pricella Biotechnology Co., Ltd., Wuhan, China) were cultured in high-glucose medium (Shanghai Dathuil Biotechnology Co., Ltd., Shanghai, China) containing 20% fetal bovine serum (FBS) and maintained in a 37 °C, 5% CO_2_ cell culture incubator. After reaching 70–90% confluency, the cells were transferred to the Lonza 4D-Nucleofector^TM^ Core Unit electroporator and subjected to the Primer Cell 3 program, with an EN150 electroporation setting. Under the 20% fetal bovine serum (FBS)-supplemented high-glucose medium (Shanghai Dathuil Biotechnology Co., Ltd.), the cells were seeded into a 6-well plate. After 24 h, cells exhibit bright green fluorescence under excitation light when observed through a fluorescence microscope. The medium was then changed to purine-containing medium (Shanghai Dathuil Biotechnology Co., Ltd.) for a 48 h drug screening, followed by replacement with complete medium containing 20% FBS.

### 2.3. In Vitro Enzyme Cleavage

The sgRNA was transcribed efficiently using the T7 RNA polymerase complex in the sgRNA in vitro transcription kit (Inovogen Tech.Co, Chongqing, China). The CRISPR/saCas9 system was subjected to in vitro enzyme cleavage experiments, CRISPR target screening, and other experiments using the in vitro enzyme cleavage kit (Inovogen Tech.Co, Chongqing, China). The synthesized sgRNA transcription templates are listed in Table S1.

### 2.4. Off-Target Analysis of sgRNA

The website (http://crispr.mit.edu/, accessed on 22 November 2023) is utilized to predict potential Off-Target Sites (OTS) for sgRNA UTY-2, and four potential off-target loci were selected from the genome. Primers designed for each off-target locus are listed in Table S1. PCR amplification was performed for each potential off-target locus, followed by Sanger sequencing to identify off-target results and determine whether mutations have occurred.

### 2.5. Screening of Monoc Slonal Cells and Junction PCR

To knock *EGFP* into the pig genome, the px459 plasmid and the targeting donor plasmid were co-transfected into cells at a ratio of 1:1.5 according to previous publication [27]. After picking clones of cell clusters under a dissecting microscope, the cells were transferred to 96-well plates. Approximately 10 days later, genomic DNA was extracted for Junction PCR identification. The PCR products were sent to BGI (Shanghai, China) for Sanger sequencing. Junction PCR primers were designed for the 5′ and 3′ ends of the integration locus, with one primer specific to the insertion sequence and the other specific to the genomic sequence outside of HMEJ. The primer sequences are provided in Supplementary Table S1.

### 2.6. Western Blot Analysis

To validate *EGFP* expression, approximately 1 × 10^6^ EGFP-positive ST cells were collected and lysed on ice for 30 min in Lysis Buffer (Sangon Biotech, China) containing proteinase and phosphatase inhibitors. Protein samples were then boiled at 100 °C for 10 min after adding protein loading buffer. Equal amounts of total protein from each sample were subjected to SDS-PAGE electrophoresis and transferred onto PVDF membranes (Servicobio, Wuhan, China). Subsequently, the membranes were blocked with 5% skim milk and incubated with GAPDH antibody (Servicobio, Wuhan, China) diluted 1:3000 and *EGFP* antibody (Servicobio, Wuhan, China) diluted 1:1000. After washing with 1×TBST, secondary antibodies, HRP-conjugated goat anti-rabbit antibody (Servicobio, Wuhan, China) diluted 1:5000 and HRP-conjugated goat anti-mouse antibody (Servicobio, Wuhan, China) diluted 1:5000, were applied. The digital signals of chemiluminescent protein blots were analyzed using the Tanon image analysis system and the software provided with the machine.

### 2.7. Quantitative RT-PCR

To identify the expression levels of the *UTY* gene in different tissues of pigs, total RNA obtained from the samples was converted into cDNA using the HiScript IV RT SuperMix for qPCR (+gDNA wiper) kit (Nanjing Vazyme Biotech Co., Ltd., Nanjing, China). Oligo7 primer analysis software was utilized for designing and evaluating primers for gene validation. The sequences of specific primers for qPCR can be found in Supplementary Table S1 (UTY-qPCR-F/UTY-qPCR-R). The relative expression levels of *UTY* between various tissues were analyzed using the QuantStudio 3 Real-Time PCR System. Taq Pro Universal SYBR qPCR Master Mix (Nanjing Vazyme Biotech Co., Ltd., China) was used for the qPCR reactions, following the manufacturer’s instructions as follows: initial denaturation at 95 °C for 10 min; followed by 40 cycles of denaturation at 95 °C for 10 s, annealing at 60 °C for 10 s, and extension at 72 °C for 15 s; and a final extension at 72 °C for 2 min, with a hold at 4 °C. *GAPDH* was used as a reference gene to normalize the expression levels. Each sample was assayed with biological triplicates, and the CT values of the *UTY* gene in each sample were detected. To detect the relative expression levels of *UTY* gene, the mRNA in the selected monoclonal positive cell lines was isolated, and then cDNA was obtained using the methods described in the above steps. The primer sequences are listed in Table S1. The differential gene expression was analyzed using the 2^−ΔΔCT^ method. Additionally, the expression of *UTY* gene across various human tissues and cells was analyzed using the Gencards database (https://www.genecards.org/, accessed on 13 April 2024).

## 3. Results

### 3.1. Identification of UTY Expression in Different Tissues

**Figure 1:**
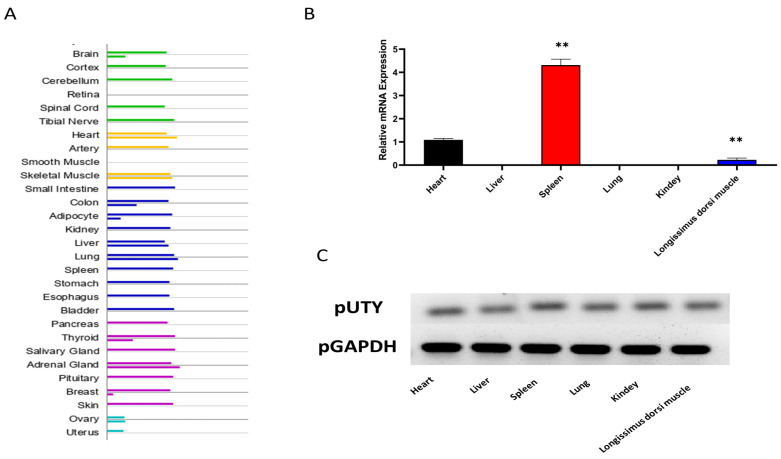
The expression of the *UTY* gene in various human and pig tissues. (**A**) The expression level of *UTY* in human tissues. (**B**) The expression of the *UTY* gene in the heart, liver, spleen, lung, kidney, and longissimus dorsi muscle of pig. (**C**) Analysis of pig *UTY* and pig *GAPDH* expression in pig organs or tissues by agarose gel electrophoresis. **: *p* < 0.01.

The expression profile of the *UTY* gene across various human tissues and cells was obtained from the GeneCards database. As showed in Figure 1A, *UTY* is widely expressed in different human tissues and cells, with a notable high expression level in pancreatic and cardiac tissues. Further investigation revealed that the expression of the *UTY* gene in various tissues of pigs was detected by real-time quantitative PCR (qPCR), including the heart, liver, spleen, lungs, kidneys and longissimus dorsi muscle. The expression of the *UTY* in the spleen and heart is significantly higher than that in other tissues (Figure 1B). The expression of pig *UTY* and *GAPDH* in the heart, liver, spleen, lung, kidney and longissimus dorsi muscle of pig was determined by agarose gel electrophoresis analysis, shown in Figure 1C.

### 3.2. The Screening of sgRNA for Porcine UTY Gene Editing

**Figure 2:**
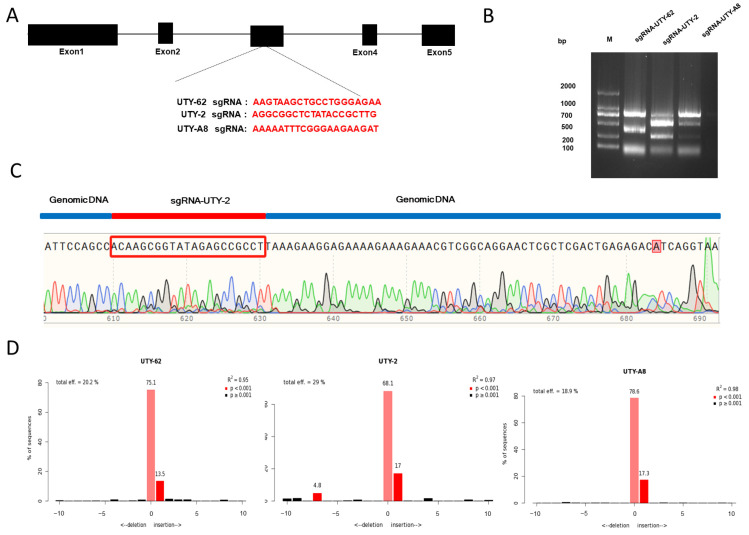
*UTY* gene-targeting schematic diagram. (**A**) The exons of *UTY* are depicted as black boxes, with the sgRNA target loci selected within the exons. The sgRNA target sequences are highlighted in red font. (**B**) An in vitro enzymatic cleavage assay kit is used to assess the activity of sgRNAs UTY-62, UTY-2, and UTY-A8; M, Marker, DL2000. (**C**) Peaks of the target locus in the sequencing curves, which are distinctly differentiated. The sgRNA UTY-2 targeted sequence is highlighted in red, while the genomic DNA is marked by the blue line. (**D**) The analysis for the indel rates of UTY-sgRNA.

The sgRNAs targeting *UTY* were designed and evaluated in pig ST cell lines using the CRISPR/Cas9 system. Three top-scoring sgRNAs were selected by conducting an sgRNA design targeting the conserved region of the *UTY* gene between the second and third exons using the online tool CCTOP website (http://chopchop.cbu.uib.no/, accessed on 6 September 2023). Based on the target loci, these three sgRNAs were connected to px459 to construct the sgRNA expression vector (Figure 2A). The plasmid sequences were validated after transformation into *Escherichia coli*, and the activity of sgRNA was confirmed before transfection. Three plasmids of PX459-UTY-sgRNAs (UTY-62, UTY-2 and UTY-A8) were separately transfected into ST cells and then genomic DNA was extracted from these cells. The efficiency of the sgRNAs was assessed through in vitro enzymatic cleavage assays according to the PCR products (The primers were listed in Table S1). The results from both the in vitro cleavage assay and the DNA sequencing confirmed the high activity of sgRNA (Figure 2B,C). The indel frequencies were calculated using the online TIDE website (https://tide.nki.nl, accessed on 15 December 2023). As showed in Figure 2D, the proportion of indels is 20.2% for UTY-62, 29% for UTY-2 and 18.9% for UTY-A8. Meanwhile, the potential OTS for UTY-2 was predicted by a website tool (http://crispr.mit.edu/, accessed on 22 November 2023). Four potential target loci (Table 1) were selected for Sanger sequencing. It was found that no mutation occurred at the potential OTS. The sequencing information for each potential OTS is provided in Figure S2.

### 3.3. Establishment of a Reporter Gene System for Genome Editing in Pig Genome

**Figure 3:**
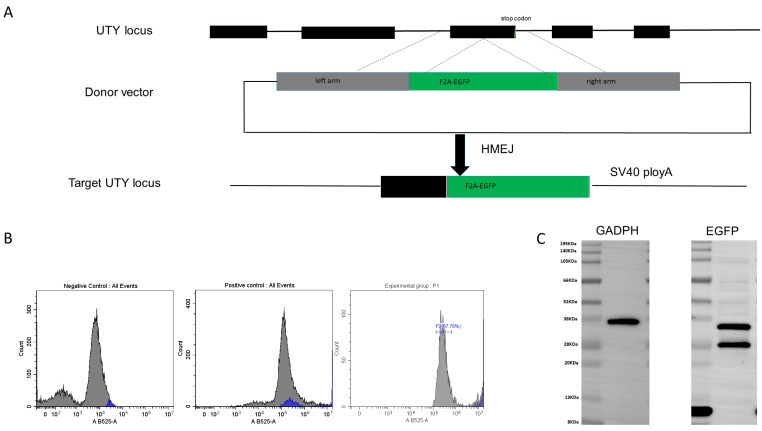
Establishment of a reporter gene system by knock-in *EGFP* at the *UTY* locus. (**A**) The exons of *UTY* are depicted as black boxes, with the stop codon shown in red boxes. The black triangular box between exon 3 and the stop codon represents the target locus of the sgRNA. A target donor is created based on the Cas9 cleavage position, carrying 800 bp of homologous regions spanning the cleavage site. (**B**) Flow cytometry analysis to count *EGFP* positive or negative cells. (**C**) Immunoblot analysis confirms the expression of *EGFP* in the pig genome.

For further research, the *EGFP* as a reporter was inserted into the *UTY* to determine whether it provides a novel safe harbor locus in the pig genome. Firstly, the donor vector carrying F2A-EGFP without a promoter, flanked by two homologous regions, was constructed. During HMEJ-mediated knock-in, the F2A-EGFP fragment was inserted into the third exon of *UTY*. Due to the presence of the self-cleaving F2A peptide, both *UTY* and *EGFP* can be expressed separately (Figure 3A). The donor plasmid and the gene-targeting vector px459 carrying the *UTY* target locus were added in a ratio of 1:1.5 for electroporation. After 48 h, the efficiency of *EGFP* gene knock-in was detected by flow cytometry, and 67.79% of the total cell was *EGFP* positive (Figure 3B). At last, the expression of *EGFP* was validated by immunoblot analysis in the pig genome (Figure 3C).

### 3.4. Analysis of Exogenous Gene Knock-In in the Pig Genome

**Figure 4:**
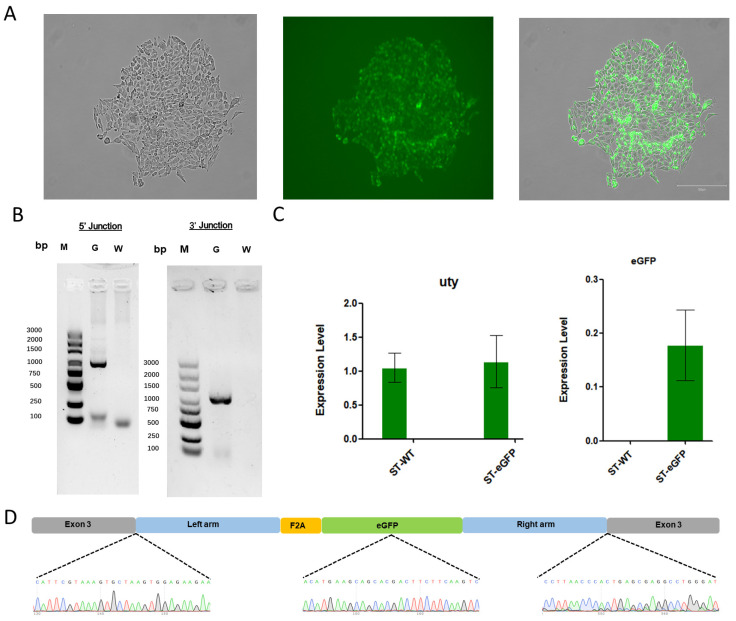
Verification of gene knock-in in EGFP-positive cells. (**A**) Representative image of *EGFP* positive ST single-cell clones. (**B**) PCR amplification of homologous arm sequences from single-cell clones. “G” lane represents the homologous arms, while “W” lane represents the negative control. M, Maker, DL2000. (**C**) The expression of mRNA for the *UTY* and *EGFP* genes, with ST-WT indicating wild-type cell lines and ST-eGFP denoting monoclonal positive cell lines. (**D**) Sanger sequencing to confirms that the exogenous gene was accurately knocked into the genome.

To achieve knock-in of the exogenous gene, the donor vector and sgRNA targeting vector were co-transfected into ST cells. These cells were cultured in medium containing puromycin for 48 h and then in medium containing 20% FBS for another 48 h. Single-cell clones were selected and cultured for another 10 d. Subsequently, single-cell clones (Figure 4A) were collected to extract genomic DNA, and primers (listed in Table S1) spanning the homologous arms were designed for Junction PCR (Figure 4B). Meanwhile, we examined the mRNA expression levels of the *UTY* and *EGFP* genes in both the wild-type cell lines and the monoclonal positive cell lines. We found that the mRNA of the *UTY* gene did not show significant changes, indicating that the integration of the exogenous *EGFP* into the *UTY* gene does not affect the expression of *UTY* itself (Figure 4C). Then, the amplified products from both sides of the homologous arms and the integrated fragment in positive cells were sequenced. The results show that exogenous *EGFP* was accurately knocked into the desired target of *UTY* on the Y chromosome (Figure 4D).

## 4. Discussion

The GSHs is a safe gene knock-in and ensures the normal and stable expression of the introduced gene. As the target integration loci for the target gene, GSHs are primarily utilized in studies of gene function, organism development, and the production of transgenic animals. Multiple safe harbor loci identified through various methods are now widely applied in different species [17,18,28,29]. This study was performed to identify and evaluate new GSH in the pig genome using CRISPR/Cas9-mediated homology arm-mediated end joining. Firstly, it was confirmed that *UTY* is expressed in pig tissues. Then, the sites at *UTY* for gene insertion were selected and the optimal sgRNA was designed and tested. The results showed that the third exon of *UTY* gene located on the Y chromosome can serve as a safe site to insert exogenous genetic materials. At last, the expression of the *EGFP* inserted into *UTY* in porcine ST cells was confirmed and its accurate locus was determined. The current evidence could be applied to generate gender-biased pigs for breeding boars producing single-sex sperm or for animal models of human diseases.

In past decades, many genetic modification methods have been successfully applied to generate transgenic or gene-edited animals with enhanced phenotypes for livestock products, disease-resistance, or pharmaceutical production [30,31,32,33]. To insert exogenous genes precisely without disrupting critical host genes, a safe harbor locus is crucial. Since 2014, the first safe harbor locus for pigs, *pRosa26*, has been identified and successfully used to generate genetically modified pigs [19]. Subsequently, *pH11*, *Pifs501*, *GAPDH* and *CKM* were tested as candidates for safe harbor loci in the pig genome [20,21,23,34]. However, these GSHs are all located on autosomes. In recent research, the sgRNA targeting the essential gene for embryonic development or gene encoding, Case 9, was inserted to the *Uty* sites of mice Y chromosome, resulting in offsprings with a single ratio [26]. Therefore, this research was designed to identify the GHS locus on the pig Y chromosome. With optimal sgRNA (sgRNA UTY-2), double-strand breaks at the *UTY* locus were efficiency induced by CRISPR/Cas9 and confirmed through in vitro cleavage analysis.

In previous research, exogenous genetic frames integration is typically achieved through HR [9,11]. The HMEJ method is an optimized version of HR, with a higher gene knock-in efficiency in various species [16]. HMEJ has shown a high efficiency in exogenous gene integration in mouse and monkey embryos, as well as in vivo liver cells and neurons, compared to strategies based on homologous recombination or non-homologous end joining [15,35,36,37]. In this study, *EGFP* was integrated at the *UTY* locus using the HMEJ method, resulting in 67.79% of pig ST cells carrying *EGFP* determined by flow cytometry analysis and further confirmed through Sanger sequencing and online TIDE analysis. Therefore, the *UTY* locus serves as a safe harbor locus for exogenous gene knock-in in the pig genome.

In the current research, the safe harbor locus of *UTY* on the pig Y chromosome has been validated. In summary, the current experiments demonstrated that the locus on the *UTY* gene is a gender-specific safe harbor locus in the pig genome. Further research focused on the long-term stability and expression of the gene inserted at the *UTY* locus should be performed. Furthermore, it can be speculated that insertion of Cas9 and sgRNA, targeting crucial genes for embryo development or spermatogenesis in the *UTY* gene, is of significant importance for sex sorting in pig breeding. This method could improve animal welfare and reduce cost, especially in the field in which animal gender is tightly associated with traits.

## 5. Patent

An application for a Chinese invention patent has been submitted for this work (application number: 202410431012.3).

## Figures and Tables

**Table 1:** Analysis of Potential OTS.

Off-Target Gene	Sequence (5′-3′)	Indel
*USPL1*	AGCCGTCT [TTATACAGCTTG] TGG	NO
*MAST2*	CGGCGGGT [CTATACCGCGGG] CGG	NO
*SPON1*	AAGCGGAT [CTGTGCCGCTTG] AGG	NO
*LARP1*	AGGCGGGC [CTTTACCCCTTG] GGG	NO

Note: Four potential OTSs were selected, and off-target results are being identified through PCR product sequencing. Blue letters represent the Protospacer Adjacent Motif (PAM) sequence. Red letters indicate differences between the sgRNA and the target sequence. The “Indel” column shows the detected off-target results.

## Data Availability

Data will be shared upon request.

## Supplementary Materials

The following supporting information can be downloaded at: https://www.mdpi.com/article/10.3390/genes15081005/s1, Table S1: Primers used in this study; Figure S1: Donor carrier mapping; Figure S2: Comparison of sequencing information of off-target sites.
