# Supplementary material for: Identification of Site in the UTY Gene as Safe Harbor Locus on the Y Chromosome of Pig
Source: Genes (Basel). 2024 Aug 1;15(8):1005. doi: 10.3390/genes15081005 (PMC11353466; doi:10.3390/genes15081005)
Supplement: Supplementary file 1 [file genes-15-01005-s001.zip › genes-3111243-supplementary.pdf]

Tables S1.

Primer	Sequence
sgRNA UTY-62-F	CACCgTTCTCCCAGGCAGCTTACTT
sgRNA UTY-62-R	AAACAAGTAAGCTGCCTGGGAGAAc
sgRNA UTY-2-F	CACCgAGGCGGCTCTATACCGCTTG
SgRNA UTY-2-R	AAACCAAGCGGTATAGAGCCGCCTC
sgRNA UTY-A8-F	CACCgAAAAATTTCGGGAAGAAGA
sgRNA UTY-A8-R	AAACATCTTCTTCCCGAAATTTTTc
USPL1-F	CCTTCTGCTCCAGGATTCTAATCT
USPL1-R	CCTAGATGCCCCCAACAAGC
MAST2-F	CCCAGAAGAGCTTCCTTCTCTTGTA
MAST2-R	GAAACATTTGTCTTCCTTCTCCCATC
SPON1-F	TCTCCTGAATTTCCTACTTCTGATC
SPON1-R	TGAAGATTAGTCCAAAGCCTCTTG
LARP1-F	TCGATTTAAGGGATGGTGGAGGG
LARP1-R	ACATTTTCTGCCCATAAAACTTGGTT
sgRNA UTY-A8 in vitro transcription	TTAATACGACTCACTATAGGGaaaaatttcgggaagaagatGTTTTAGAGCTAGAAATA
sgRNA UTY-62 in vitro transcription	TTAATACGACTCACTATAGGGaagtaagctgcctgggagaaGTTTTAGAGCTAGAAATA
SgRNA UTY-2 in vitro transcription	TTAATACGACTCACTATAGGGccacaagcggtatagagccgGTTTTAGAGCTAGAAATA
Left-Junction PCR-F	CCATAGGATTAGGTTCCCTATTTATGCCAT
Left-Junction PCR-R	CTGGACCTGGATTGCTTTCTACATCC
right-Junction PCR-F	CATAATCAGCCATACCACATTTGTAGAGGTTTT
right-Junction PCR-R	CAACAACAGTACGATTACCATTTAGGTCAGTAT
qPCR-UTY-F	CCTTACATGAGGAGTGGTTGTTGAG
qPCR-UTY-R	TCTTCCCATTCTTCCCTTAACTTTCG
qPCR-GAPDH-F	AGGCTGTGGGCAAGGTCATC
qPCR-GAPDH-R	CAGGCGGCAGGTCAGATCC
qPCR-eGFP-F	ACCCTCGTGACCACCCTGAC
Qpcr-eGFP-R	TGTAGTTGCCGTCGTCCTTGAAG
UTY-F1	GTCACAAGACACACAATACAGCGAT

UTY-R1	ACCTGAGTCTCTCAGTCGAGC

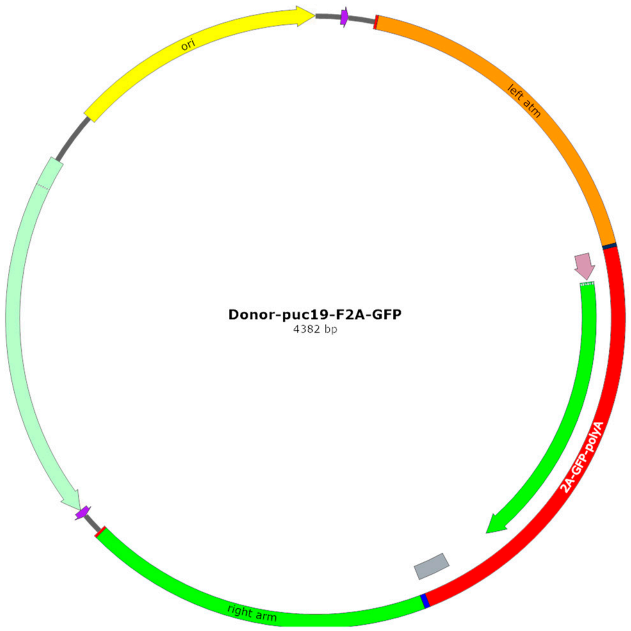

Fig.S1: Donor carrier mapping

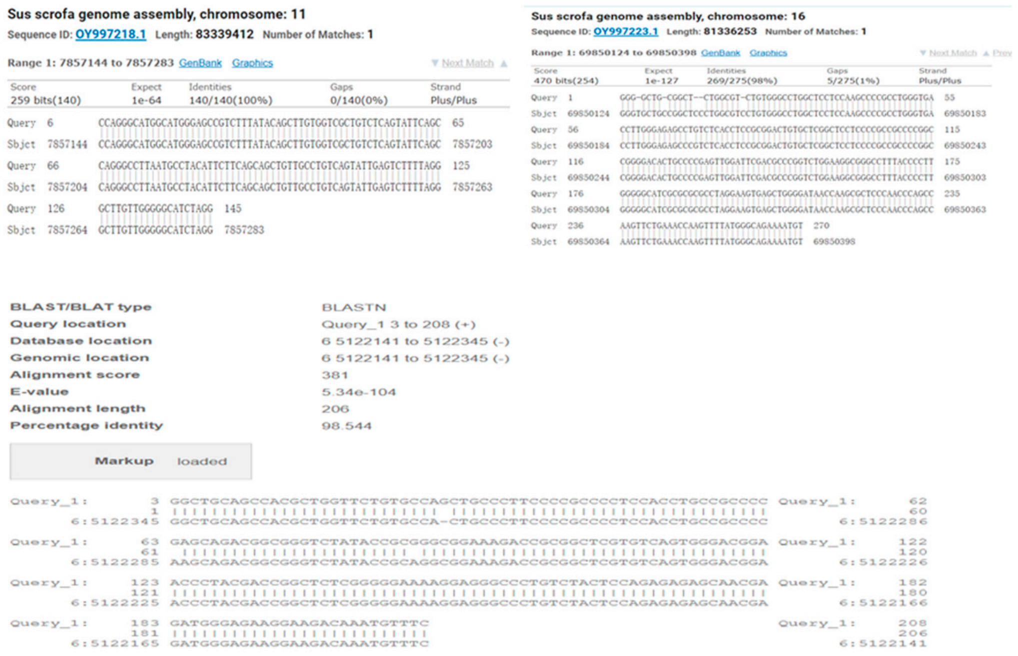

Fig.S2:Comparison of sequencing information of off-target sites
